# Assessing Edible Filamentous Fungal Carriers as Cell Supports for Growth of Yeast and Cultivated Meat

**DOI:** 10.3390/foods11193142

**Published:** 2022-10-09

**Authors:** Minami Ogawa, Jaime Moreno García, Nitin Nitin, Keith Baar, David E. Block

**Affiliations:** 1 Department of Food Science and Technology, University of California, Davis, Davis, CA 95616, USA; 2 Department of Agricultural Chemistry, Edaphology and Microbiology, University of Córdoba, 14014 Córdoba, Spain; 3 Department of Neurobiology, Physiology and Behavior, University of California, Davis, Davis, CA 95616, USA; 4 Department of Viticulture and Enology, University of California, Davis, Davis, CA 95616, USA; 5 Department of Chemical Engineering, University of California, Davis, Davis, CA 95616, USA

**Keywords:** filamentous fungus, edible carrier, adherent cell, yeast immobilization, cultivated meat

## Abstract

The growth and activity of adherent cells can be enabled or enhanced through attachment to a solid surface. For food and beverage production processes, these solid supports should be food-grade, low-cost, and biocompatible with the cell of interest. Solid supports that are edible can be a part of the final product, thus simplifying downstream operations in the production of fermented beverages and lab grown meat. We provide proof of concept that edible filamentous fungal pellets can function as a solid support by assessing the attachment and growth of two model cell types: yeast, and myoblast cells. The filamentous fungus *Aspergillus oryzae* was cultured to produce pellets with 0.9 mm diameter. These fugal pellets were inactivated by heat or chemical methods and characterized physicochemically. Chemically inactivated pellets had the lowest dry mass and were the most hydrophobic. Scanning electron microscope images showed that both yeast and myoblast cells naturally adhered to the fungal pellets. Over 48 h of incubation, immobilized yeast increased five-fold on active pellets and six-fold on heat-inactivated pellets. Myoblast cells proliferated best on heat-treated pellets, where viable cell activity increased almost two-fold, whereas on chemically inactivated pellets myoblasts did not increase in the cell mass. These results support the use of filamentous fungi as a novel cell immobilization biomaterial for food technology applications.

## 1. Introduction

Whole-cell immobilization technology aims to attach intact cells to a support material that supports cell growth and differentiation [1]. This system of immobilizing cells to a carrier can be advantageous over freely suspended cells because immobilization frequently facilitates an increase in metabolic product yield, product recovery, cell stability, cell recovery, and reactor performance during fermentation, as well as allowing suspended culture for adherent cells [2,3,4]. The discovery of novel materials that support the growth and differentiation of cells is crucial, especially for the sustainable food technology sector, where food grade requirements must be met. An ideal edible support material must have favorable chemical and mechanical stability, wide applicability, high biocompatibility, and low-cost [2,3,4,5,6].

Several emerging biomaterials have been developed in recent years for cell immobilization support that meet some or all of these criteria [7,8,9,10]. Filamentous fungal biomass is one of these promising technologies as it can be edible and is produced through a food-grade fermentation process. By utilizing GRAS fungal strains such as *Aspergillus oryzae*, the fungal support can be consumed whole, as an integral part of a food product, and therefore has applications in the beverage and food production sectors. One of the most defining features of filamentous fungus (FF) is its ability to grow quickly on simple, low-cost carbon sources and several FF species are able to spontaneously form spherical masses, called pellets [11]. Pellets are a matrix of intertwining hyphae and have a hollow or sparse interior core [12]. From an immobilization perspective, the pellet geometry is beneficial because the hyphae layer is dense and flexible enough to maintain structure and withstand high mechanical forces to provide a robust platform for cell attachment while having a porous wall that allows mass transfer of nutrients, high surface-area-to-volume ratio, and low overall density. These properties allow pellets to remain mechanically resilient while suspended in bioreactors under gentle agitation [13]. Structurally, the fungal cell wall is composed of layers of proteins, glucans, and chitin, which not only gives FF high-protein and high-fiber nutritional value but provides anchorage points to which flocculating cell receptor ligands can adhere [14]. Because of these characteristics, fungal pellets can be an ideal cell immobilization platform that is scalable and useful for large-scale fermentation and cultivation practices.

In this experiment, we explore two different types of cells immobilized onto FF as a support: *Saccharomyces cerevisiae* yeast and C2C12 murine myoblast cells. These two cell types were selected to provide insight into the factors that impact cell attachment and growth with specific application to food industry sectors. Yeast immobilization to fungal biomass has been studied previously and is utilized for alcoholic fermentation such as for the production of beer, wine, and bioethanol [15,16,17]. The attachment of yeast to FF is termed a yeast biocapsule. Yeast biocapsulation improves cell stability; allows for continuous operation, operational flexibility, and control of fermentation; and facilitates cell recovery and reuse [3,18]. In the past, yeast biocapsule formation required careful control of fermentation conditions and long incubation times for both the FF and yeast to remain viable. The method proposed in this study looks to accelerate biocapsule formation by adhering yeast to already inactivated fungal pellets, a technique that also improves the quality of biocapsule formation.

The second novel application of fungal pellet supports is as edible microcarriers in cultivated meat production. Edible filamentous fungal supports may be able to replace conventional non-edible microcarriers, which are used to produce high cell density for anchorage-dependent animal cells. Since traditional microcarriers are nonedible and need to be removed from the final product to meet taste and sensory requirements, there are extensive downstream operational costs associated with the dissociation of the meat cells from the carriers, the separation of the meat cells, and the elimination of microcarriers [19]. Edible filamentous microcarriers, by contrast, could remain an integral part of the final product, simplifying downstream processing and formulation and providing opportunities to improve the nutritional and/or organoleptic qualities of the final product. In muscle cell cultivation, the FF edible support would need to be inert to avoid interference with the cultivation and growth of the more slowly growing myoblasts.

In both applications, cell attachment is the critical first step in assessing the validity and efficiency of the immobilization technology. Low attachment efficiency would lead to low productivity [20]. Support surface properties can impact cell attachment [3,14,19]. These properties include chemical traits such as the charge or hydrophilicity, and physical attributes such as the size, shape, and topography of the carrier. In this study, we grow fungal pellets and inactivate them using conventional sterilization techniques common in the food sector. The active and inactive pellets are then characterized, and the impact of these properties on cell attachment are assessed using *S. cerevisiae* yeast and C2C12 myoblast cells as a proof of concept. In the case of myoblast cells, only inactive fungal pellets were studied to provide an inert support, and yield was compared with a nonedible microcarrier, Cytodex 3. The activity of both model systems post-attachment was also assessed. With this study, we test whether we can use established scalable fermentation processes to produce edible fungal pellets to support the growth of adherent cells for cultivated meat and fermentation practices.

## 2. Materials and Methods

### 2.1. Cells and Media Components

The FF used in this study was *A. oryzae* UCD 76-2 (UC Davis Phaff Culture Collection, Davis, CA, USA), selected for its GRAS status and the history of its biomass consumed and used as a food source [21]. The FF was grown on sporulation agar plates (1.7% (*w*/*v*) corn meal agar (BD, Franklin Lakes, NJ, USA), 0.1% (*w*/*v*) yeast extract (BD, Franklin Lakes, NJ, USA), 0.2 % (*w*/*v*) glucose (Spectrum Chemicals, New Brunswick, NJ, USA) and 2 % (*w*/*v*) bactoagar (BD, Franklin Lakes, NJ, USA)) and FF pellet medium (6% (*w*/*v*) glucose, 0.3% (*w*/*v*) yeast extract, 0.3% (*w*/*v*) NaNO_3_ (Sigma-Aldrich, St. Louis, MO, USA), 0.1% (*w*/*v*) K_2_HPO_4_ (Thermo Fisher Scientific, Waltham, MA, USA), 0.05% (*w*/*v*) MgSO_4_, (RPI, Mount Prospect, IL, USA) 0.05% (*w*/*v*) KCl (Thermo Fisher Scientific, Waltham, MA, USA), and 0.001% (*w*/*v*) FeSO_4_ (Thermo Fisher Scientific, Waltham, MA, USA). The yeast *S. cerevisiae* G1 (ATCC: MYA-2451) (UC Davis Viticulture and Enology Culture Collection and University of Cordoba, Spain) was selected because of its high affinity to FF in previous yeast immobilization studies [18]. The yeast was pre-grown on YPD agar (yeast extract 1% (*w*/*v*), peptone 2% (*w*/*v*), glucose 2% (*w*/*v*), and bacto-agar 2% (*w*/*v*)) and YPD liquid. C2C12 cells (mouse myoblasts; ATCC: CRL-1772) were used for the animal cell immobilization and cultivated in complete proliferation media: 89% (*v*/*v*) DMEM (Thermo Scientific, Rochester, NY, USA) 10% (*v*/*v*), fetal bovine serum (Thermo Scientific, Rochester, NY, USA), and 1% (*v*/*v*) Penicillin-Streptomycin (Sigma-Aldrich, St. Louis, MO, USA).

### 2.2. Fungal Pellet Formation and Inactivation

FF was pre-grown on sporulation agar plates for 7 days at 28 °C. Spores were then suspended in sterile distilled water, the suspension vortexed and sonicated for 5 min, and a volume inoculated to reach a final concentration of 1 × 10^6^ spores/mL in 50 mL of FF pellet medium with pH adjusted to 5.5 in 250 mL Erlenmeyer flasks covered with hydrophobic cotton. The flasks were incubated under high agitation (250 rpm at 30 °C). After 3 days, the active fungal pellets (AFP) were harvested and washed with sterile DI water. For inactive pellet conditions, heat-treated fungal pellets (HFP) were autoclaved in sterile DI water at 121 °C for 20 min and chemically treated fungal pellets (CFP) were placed in 50 mL of 70% (*v*/*v*) ethanol in 250 mL flask and incubated for 20 min at 100 rpm and washed with sterile DI water. Both inactivation methods were performed immediately after pellet formation was complete. The viability of pellets was confirmed by taking single pellets, assessing the growth on YPD plates, and incubating for 5 days at 28 °C. Each viability check was repeated with 12 randomly chosen pellets.

### 2.3. Measurement of Pellet Parameters

After the formation of active and inactivated pellets, individual pellet mass was measured by wet weight and dried in a 100 °C oven overnight to obtain the dry weight. The diameters of pellets were measured in their wet state from digital images using the particle analyzer feature in ImajeJ2 software 2.3.0 (Bethesda, Rockville, MD, USA) [22]. The volume of pellets was measured by measuring displacement in volume after and before submersion for 1 mL of settled pellets. Further, some other traits that influence cell immobilization were quantified: charge, elemental composition, and hydrophobicity. The charge of the pellets was analyzed using a Zetasizer (Malvern Panalytical, Worcestershire, UK). Since this instrument measures particle sizes of up to 100 microns, pellets were mechanically broken into smaller hyphae fragments using a homogenizer (IKA, Staufen, Germany) at a 14 k setting for 3 min. The mixture was filtered using a 300 micron filter and subsequently through an 80 micron filter to remove larger particles, and the remaining suspension was measured for zeta potential. Carbon percentage was analyzed by Thermo Fisher Quattro S. Environmental scanning electron microscope (Thermo Scientific, Rochester, NY, USA) equipped with a QUANTAX EDS detector (Bruker, Billerica, MA, USA). The hydrophobicity of the pellets was measured using a phase-partition method described by Rosenberg et al. [23]. Briefly, pellets were washed with phosphate buffer saline (PBS), and 0.5 mL of settled pellets was placed in 9.5 mL of PBS. The optical density of the cell suspension was measured at 640 nm (*A*1) using a Genesys 10 s UV-Vis spectrophotometer (Thermo Scientific, Rochester, NY, USA). Next, 1.4 mL of octane (Alfa Aesar, Haverhill, MA, USA) was added to the 10 mL of fungal suspension, mixed with a vortex mixer for 2 min, and allowed to stand for 10–15 min until phase separation was observed. The upper hydrocarbon phase was discarded, and the lower aqueous phase was collected and measured for optical density at 640 nm (*A2*). The hydrophobicity index (HPBI) was determined as

(1) $$\mathrm{HPBI}=\frac{A1-A2}{A1}\times 100$$

### 2.4. Measurement of Yeast Cell Immobilization Potential

*S. cerevisiae* G1 was pre-grown on YPD agar, and a colony was inoculated in 50 mL of YPD liquid medium in a 250 mL flask at 175 rpm, 28 °C for 3 days. The suspension was centrifuged for 10 min at 4200 rpm and the supernatant discarded. Yeasts were weighed and combined with AFP, HFP, or CFP at a weight ratio of 1:10 in a sterile container. YPD liquid was added to the same container to submerge pellets and gently shaken to homogenize the solution. The container was left loose-capped, and yeasts were left to grow for 72 h at 28 °C under static condition. At this point, the yeast cells began attaching to fungal mycelium to form the yeast-fungus structure referred to as yeast biocapsules, which can be applied to alcoholic fermentation production such as beer, wine, and bioethanol. Once the yeast biocapsule incubation was finished, the supernatant was decanted and yeast biocapsules were washed twice with sterile DI water.

Samples were taken at 24 h and 72 h, and the immobilization yield was counted directly after sampling. For the immobilized yeast counting, ten biocapsules out of the total from each flask were disrupted with salt to separate yeast cells from the fungal hyphae. Biocapsules were broken by placing them into a NaCl solution (100 mM), grinding them with a tissue grinder for 2 min, and then transferring them to a test tube and sonicating for 20 min. As a result, a mixture of yeast cells and fungal hypha segments was obtained. The immobilized yeast number was determined by direct counting using a hemacytometer grid under the microscope at 40× objective.

### 2.5. Microscopic Imaging Analysis

To analyze the fungal pellet structure, pellets were observed using a Thermo Fisher Quattro S Environmental scanning electron microscope (Thermo Scientific, Rochester, NY, USA) in high vacuum mode using 5 kV accelerating voltage to capture pellets before a brief increase to 15 kV for imaging. Prior to imaging, pellets and yeast biocapsules were freeze-dried and gold-sputter-coated. To observe the inner area of the fungal pellets or yeast biocapsules, pellets were cut in half with a scalpel. Yeast cells in images obtained from SEM were later colored using Adobe Photoshop CS6 13.1.3 by Adobe Inc. (San Jose, CA, USA).

### 2.6. Measurement of Animal Cell Immobilization Potential

C2C12 cells were immobilized on fungal pellets and Cytodex 3 (Cytiva, Marlborough, MA, USA), a nonedible conventional microcarrier. Cytodex 3 was used as a positive control and prepared prior to seeding according to the manufacturer’s instructions. Fungal microcarriers (HFP and CFP) were prepared by placing 20 inactivated pellets in each well of a 96 well plate (Corning, Somerville, MA, USA), washing twice with DPBS, and submerging them in complete proliferation media for 15 min at 37 °C with 5 % CO_2_. AFP were not tested, with the reasoning that fungal metabolism will outcompete the animal cell metabolism and prevent animal cell growth. C2C12 cells were seeded at 3000 cells/cm^2^ in 96 well plates coated with anti-adherent solution (StemCell, Vancouver, WA, Canada) to prevent cell attachment to the bottom of the wells. No microcarriers were placed in negative control wells, and a second positive control, cells growing on a standard tissue culture-treated 96 well plate that promotes cell adhesion, was run in parallel. All wells contained 200 μL total of complete proliferation media, incubated at 37 °C, 5% CO_2_ and assessed at 24 h and 72 h for cell viability. AlamarBlue^®^ Cell Viability Assay Reagent (AB) (Invitrogen, Waltham, MA, USA) was used to quantify cellular metabolic activity and in turn determine the concentration of viable cells in a given sample. Briefly, the dye incorporates an oxidation-reduction (REDOX) indicator that both fluoresces and changes color in response to chemical reduction within active mitochondria. In total, 20 μL of AB was administered in each well at each time point. After 4 h, the absorbance of each well was measured at 570 nm and 600 nm with SpectraMax iD5 Multi-Mode Microplate Reader (Molecular Devices, San Jose, CA, USA). The percent reduction of AB was calculated using Equation (2).

(2) $$\mathrm{Percent}\;\mathrm{reduction}\;\mathrm{of}\;\mathrm{AB}=\frac{\left(O2\times A1\right)-\left(O1\times A2\right)}{\left(R1\times N2\right)-\left(R2\times N1\right)}\times 100$$

where *O*1 is the molar extinction coefficient (E) of oxidized AB at 570 nm, *O*2 is the E of oxidized AB at 600 nm, *R*1 is the E of reduced AB at 570 nm, *R2* is the E of reduced AB at 600 nm, *A*1 is the absorbance of test wells at 570 nm, *A2* is the absorbance of test wells at 600 nm, *N*1 is the absorbance of negative control well (media plus microcarriers and AB but no cells) at 570 nm, and *N*2 is the absorbance of negative control well at 600 nm. The higher the percentage reduction of media, the more metabolically active cells are present.

### 2.7. Statistical Analysis

Statistical analysis was performed using the software package Statgraphics^®^ Centurion XVI from Stat Points Technologies, Inc. (Warrenton, VI, USA) for the following methods: ANOVA (Analysis Of Variance) and Fisher test for the establishment of Homogeneous Groups (HG) at a significance level *p* ≤ 0.05. Standard deviation was calculated for all values for pellet diameter, pellet number, and cell immobilizations.

## 3. Results

### 3.1. Fungal Pellet Growth and Characterization

**Figure 1:**
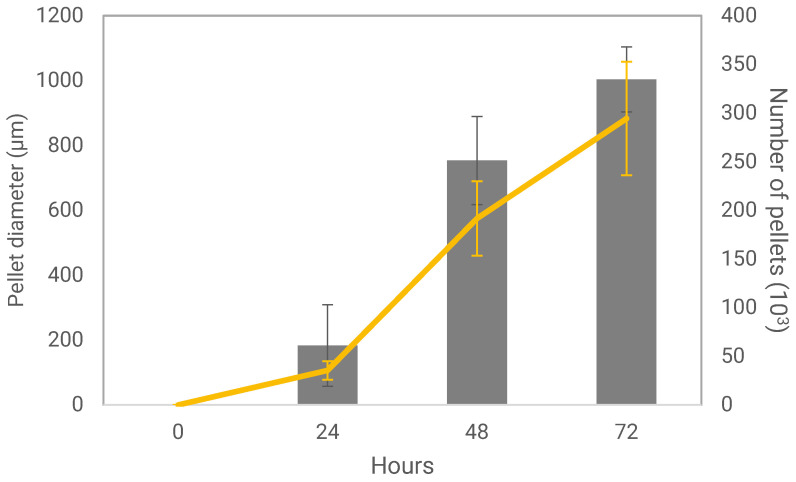
Pellet diameter and number of pellets per flask measured over the course of 72 h from the point of inoculation. The bars represent the number of pellets, and yellow line represents pellet diameter.

**Figure 2:**
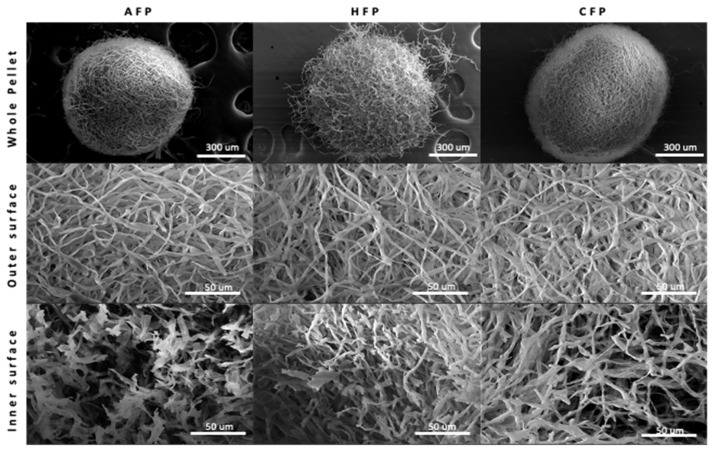
SEM of whole pellet, outer surface, and inner surface of fungal pellets. Active fungal pellets (AFP), heat-treated fungal pellets (HFP), and chemically treated fungal pellets (CFP).

Fungal pellets were first produced in flasks in order to characterize their growth and final properties. Fungal pellets were grown over the course of 72 h, during which time the pellet diameter and number of individual pellets per flask increased, particularly between 24 and 48 h (Figure 1). Inactivation was achieved by HFP at 121 °C for 20 min or CFP with 70% (*v*/*v*) ethanol for 20 min. Following inactivation, the physiological characteristics of fungal pellets did not change significantly when compared to the AFP. Pellets retained their spherical shape and diameter (Figure 2 and Table 1), even after inactivation treatments. All pellets appear to have a compact spherical structure, with a clearly defined outer wall containing hyphae that protrude from the surface in HFP. In all conditions, the outer surface of the pellets is microtextured with intertwining layers of fungal hyphae with an individual diameter of 2 to 3 microns. The void spaces between hyphae are present in within the pellets, emphasizing the porous nature of the pellet geometry.

Other measured parameters, which were significantly different between the activated and inactivated fungal pellet conditions, were (1) the dry mass, where AFP was 0.4 ± 0.01 mg/fungal pellet compared to HFP with 0.3 ± 0.01 mg/fungal pellet and CFP 0.01 ± 0.01 mg/fungal pellet; (2) the hydrophobicity, with HPBI varying from AFP at 4.7 ± 0.1, to 2.1 ± 0.5 and 0.7 ± 0.1 in the HFP and CFP, respectively (Table 1); and (3) the charge of the pellet (AFP had a stronger negative charge compared to the inactivated HFP and CFP; however, all conditions presented zeta potentials that are fairly close to neutral). The carbon composition of fungal pellets did not show significant differences: AFP was 61 ± 10%, HFP was 53 ± 9%, and AFP was 50 ± 8% carbon by mass. Materials with high carbon content or carbonaceous material can improve cell activity and growth and assist interspecies electron transfer, buffering capacity, and nutrient adsorption onto their surface [24,25]

### 3.2. Yeast Cell Immobilization

**Figure 3:**
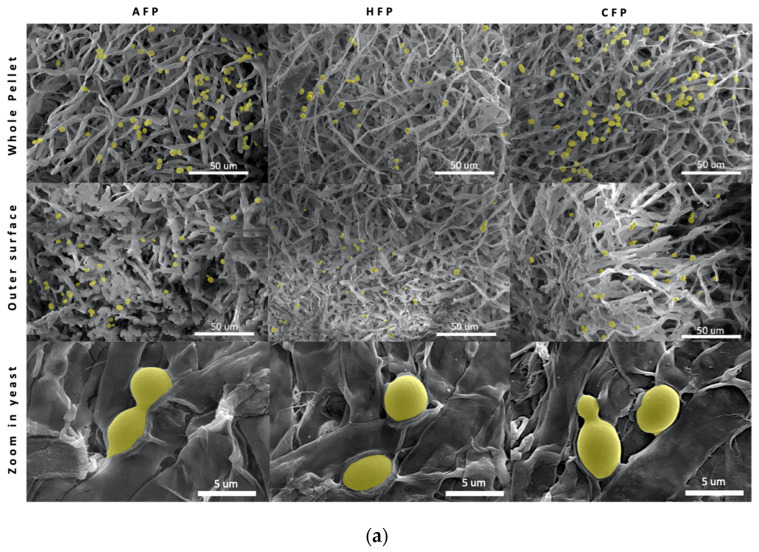

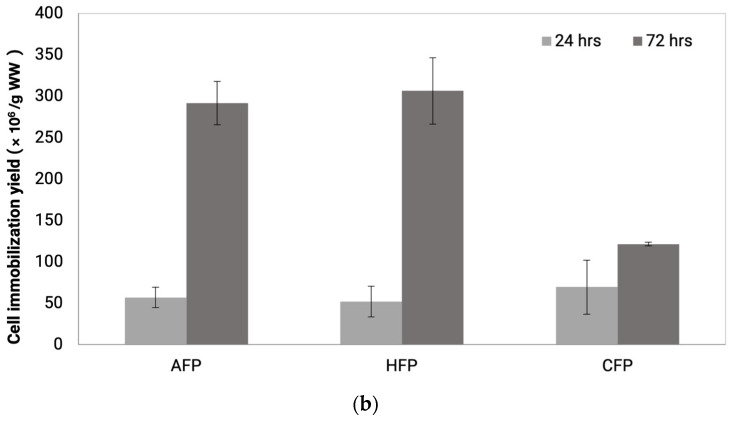
(**a**) SEM of yeast biocapsules with yeast cells (colored in yellow manually) on the outer surface and inner surface of fungal pellets. Yeasts immobilized on active fungal pellets (AFP), heat-treated fungal pellets (HFP), and chemically treated fungal pellets (CFP). (**b**) Cell immobilization yield at 24 h and 72 h incubation.

The immobilization of yeast cells onto fungal pellets was confirmed both by direct counting of yeast and by SEM imaging. In the SEM images, yeast cells can be seen attached both on the outer surface of the pellet and the inner area of the pellets, with more cells adhering to the outer surface compared to the inner (Figure 3a). At higher magnification, it is clear that the yeast cells are firmly attached to the hyphae walls, as if they are embedded into the fungal matrix. Some yeasts bud and show signs of replicating while remaining adhered onto the hyphae. The newly formed daughter cells may be using the same natural process of adhesion as the parental cells. This observation is consistent with previous literature where authors saw an increase in immobilized cells after the yeast biocapsules were incubated in a nutrient rich media [26].

The yeast immobilization yields, or yeast cell mass, increased over time for all conditions, where AFP and HFP showed a higher increase in yield from 24 h to 72 h incubation compared to CFP. AFP increased from 56.8 ± 12.3 × 10^6^ cells/g wet weight to 291.8 ± 26.2 × 10^6^ cells/g wet weight, HFP increased from 51.0 ± 18.5 × 10^6^ cells/g wet weight to 306.4 ± 40.1 × 10^6^ cells/g wet weight, and CFP increased from 69.4 ± 32.6 × 10^6^ cells/g wet weight to 121.3 ± 2.2 × 10^6^ cells/g wet weight (Figure 3b). The decreased immobilization efficiency of CFP may be related to the loss of hydrophobicity since hydrophobic cell surface components that may be important in yeast-FF attraction were lost with ethanol treatment.

### 3.3. Animal Cell Immobilization

**Figure 4:**
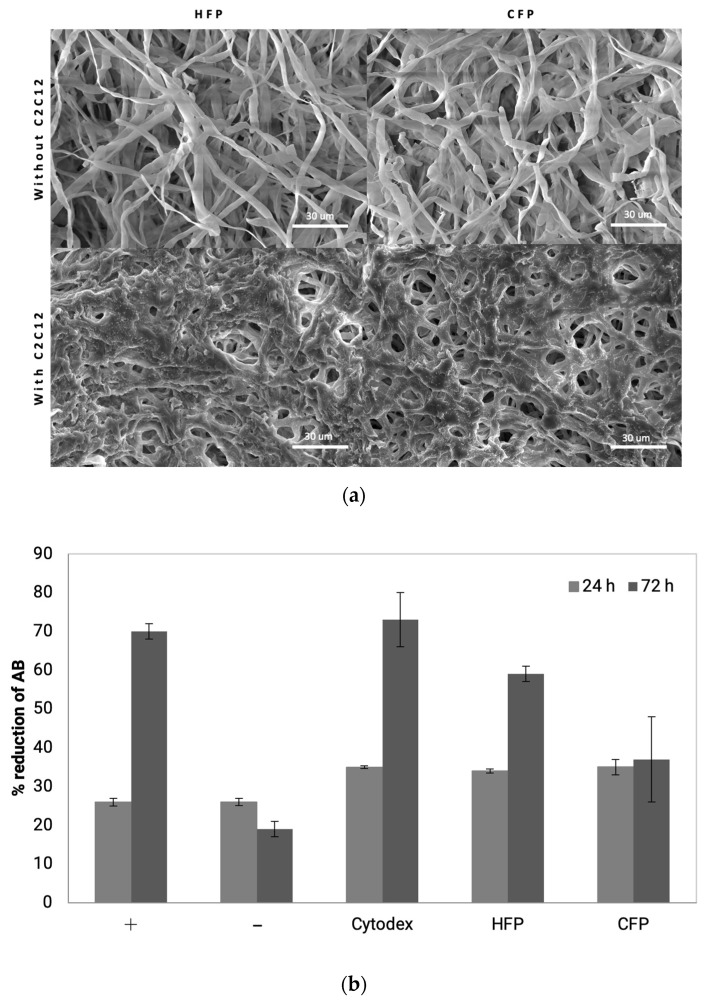
(**a**) SEM of raw fungal pellet surface and with C2C12. (**b**) Viability of C2C12 on microcarriers using percent reduction of alamar blue (AB) as an indicator of cell number. Light grey bars are samples at 24h incubation and dark grey bars are 72h incubation after seeding. Positive control is C2C12 grown on tissue culture treated plates, negative control is C2C12 forced to grow in suspension on low attachment plates, HFP are C2C12 cells cultivated on heat-treated fungal pellets, and CFP are C2C12 cells cultivated on chemically treated fungal pellets.

C2C12 murine myoblast cells were used to study animal cell attachment and growth on fungal pellets as an edible carrier. The attachment of C2C12 to microcarriers was visualized by SEM images (Figure 4a), where a mass of cells can be seen to cover the surface of both CFP and HFP pellets. C2C12 cells are anchorage-dependent, in other words, they require attachment to an inert surface in order to remain viable and proliferate. This was demonstrated in the negative control conditions, where C2C12 cells were seeded in wells coated with an anti-adherent solution and no microcarriers. When forced to remain in suspension, no growth was observed between 24 and 72 h (Figure 4b). In contrast, the positive control, where cells were seeded on tissue culture treated plates, proliferated, as indicated by the increase in AB reduction from 26 ± 1% to 70 ± 2% in 72 h. Cytodex 3 showed similar growth to the positive control, showing an increase from 35 ± 0.3% to 73 ± 7% over the same time period. The fungal pellets showed slightly different proliferation trends depending on the treatment. CFP exhibited no growth from 24 to 72 h going from 35 ± 2% to 37 ± 11% reduction. On the other hand, on HFP myoblasts went from 34 ± 0.5% to 59 ± 2% reduction in AB, indicating growth or increased metabolic activity comparable to the positive controls. These data give a clear indication that heat-treated fungal pellets could be a viable edible microcarrier for the scaling up of cultivated meat relevant cell lines.

## 4. Discussion

This study provides a proof of concept that inactive fungal pellets can function as a support for cell attachment and growth, with the focus placed on two model cell types, *S. cerevisiae* and the C2C12 muscle cell line. We were successfully able to culture edible FF to achieve small pellet sizes, confirming the method reported by Jeenor er al [27]. In the same study, Jeenor et al. [27] reported the scalability of the method by culturing *A. oryzae* in bioreactors with a 5 L capacity. Fungal pellet fermentation was also performed at a commercial scale to produce industrially relevant enzymes and compounds [12,28,29]. Our aim was to utilize an existing scalable process, to determine whether these pellets have the potential to be used for yeast or animal cell adhesion and growth. The development of edible microcarriers would reduce the number of operations and downstream processing and improve the economics of cellular agriculture by reducing raw material cost/unit operation. We were able to validate fungal microcarriers for cell culture by confirming cell attachment and the growth of cells on the engineered pellets. For the animal cells, these data suggest edible fungal pellet microcarriers are a viable path to creating animal cell mass at commercial scale.

Following inactivation, there were no significant differences physically, such as pellet size and surface texture of the pellets; however, dry weight and hydrophobicity varied with inactivation technique. Dry weight was the lowest in the CFP group, which may be explained by action of ethyl alcohol on the pellets. Because ethyl alcohol is amphiphilic, it denatures proteins and solubilizes lipids and hydrophobic proteins, such as oxylipins and hydrophobins [30]. The result is damage to cell membrane and cell wall components, followed by the solubilization of cytosolic components by the aqueous part of the solution and a reduction in dry weight of the fungal biomass. HFP are inactivated by autoclaving. Autoclaving causes irreversible coagulation and the denaturation of enzymes and structural proteins by quickly raising the heat to 121 °C using saturated steam at 1 atm overpressure [31]. This caused FF surface properties such as hydrophobicity and charge to decrease; however, the mass of the pellets was not significantly affected.

For yeast cell immobilization, yeast were seen to immobilize both on active pellets and inactive pellets. In prior studies, yeast immobilization on mycelium occurred by co-culturing FF and yeast, where both microorganisms remained viable. Using this method, the growth of both FF and yeast was observed simultaneously in liquid culture over the course of 7 days, resulting in the immobilization of 200 × 10^6^ yeast cells/g wet weight. These results match previous studies comparing yeast biocapsules to alginate beads, the conventional yeast immobilization system, where yeast biocapsules had similar or higher yeast density per wet weight [32]. By contrast, in the current study, we observed the direct attachment of yeast to already formed filamentous fungal pellets, and similar yeast immobilization was achieved after 3 days of incubation. Further, in order to inactivate only the FF while keeping yeast alive, prior methods required an additional 12-day cycle of incubation in high-sugar medium (YP + 25% dextrose), which caused the yeast to multiply, ferment, and produce ethanol concentrations high enough to inactivate the FF but not the yeast.

A novel finding of the current work is that yeast cell growth was lower on CFP compared to the other conditions. Yeast cell attachment is mediated by cell surface proteins, called flocculins, that directly participate in adhesion of cells to each other or other substrates [14,33]. The flocculins recognize and bind to α-mannan residues (receptors) of neighboring cells and other flocculins embedded in the rich outer mannoprotein layer of the cell wall [34,35]. As described earlier, ethyl alcohol treatment may denature these receptors on the FF, leaving fewer anchorage points and limiting binding to a peak at 24 h of incubation [36]. Further, the CFPs were also significantly less hydrophobic than the other conditions, most likely due to the solubilization of fatty acid components and the removal of hydrophobins. Hydrophobicity plays a part in yeast-FF attraction since flocculation proteins responsible for yeast attachment have hydrophobic domains and are attracted to other hydrophobic surfaces. The loss of hydrophobicity may therefore have contributed to the decrease in yeast cell immobilization yield for CFP.

The C2C12 muscle cell line also exhibited attachment and growth on fungal pellets, with HFP showing comparable growth to the commercial Cytodex 3, the conventional cell immobilization technology for in vitro animal cells [37]. Most conventional microcarriers such as Cytodex 3 are composed of non-edible materials such as cross-linked dextran and coated with animal derived ECM such as pig skin gelatin [38]. Even though the fungal pellets were not coated with specific ECM proteins, cell attachment and viability were still observed. The physicochemical properties of the fungal pellets were sufficient to promote cell attachment and allow immobilization yields comparable to Cytodex 3 microcarriers. The fungal pellets are four times larger than Cytodex microcarriers. Schmidt et al. [39] reported that microcarrier size affects cell behavior, where microcarriers between 1500 and 3000 μm promote cell attachment compared to smaller 500 μm microcarriers. Fungal pellets also have microtextured surface typology. C2C12 cells have been shown to adhere better to micropatterned surfaces because they more closely resemble the native ECM [40]. Interestingly, HFP showed increased C2C12 viability and metabolic activity compared to CFP despite the size and surface typology remaining similar. The difference may be attributed to the difference in pellet stiffness, which has been reported to be a crucial parameter for cell attachment affecting protein expression, cytoskeleton modification, and cell viability [41,42,43]. The change in dry weight in the absence of a change in pellet size as a consequence of inactivation treatments suggests that the density (or stiffness) of fungal pellets was modified. While these characteristic changes may impact cell attachment, there are also reports that proliferation, alignment, and fusion are promoted on microcarriers that are smaller and have smoother surfaces [39,40]. In order to assess the full efficacy of cell growth on fungal pellets, longer proliferation and differentiation studies are required. Nonetheless, the data from this study confirms mammalian cell attachment and growth on FF pellets, a prerequisite for further investigation as an edible microcarrier for cultivated meat.

## 5. Conclusions

The data presented in this study demonstrate that inactive fungal biomass can be used to attach and grow very different cell types and is a proof of concept of tunable and edible fungal pellets for cellular agriculture. In future experiments, cell attachment can be enhanced by modifying the fungal pellet, changing fermentation conditions, utilizing different species/strains of FF, or engineering FF with other desired properties. It should also be possible to introduce qualities that not only improve cell immobilization but boost the flavor, taste, and nutritional properties of the final product, for example, studying the change in aromatic profiles when using yeast biocapsules for alcoholic fermentations or the effect on texture and taste when fungal microcarriers are incorporated into cultivated meat products. The data presented in this study unlock the potential of fungal pellet technology and serve as a foundation for future cell immobilization studies.

## 6. Patents

The work reported in this manuscript resulted in U.S. Provisional Patent Application No. 63/187,178, filed 11 May 2021 and International Patent Application No. PCT/US2022/028637 filed 10 May 2022.

## Figures and Tables

**Table 1:** Characteristics of fungal pellets for active fungal pellets (AFP), heat-treated fungal pellets (HFP), and chemically treated fungal pellets (CFP). Superscript letters (a–c) indicate statistically significant homogenous groups differing in the parameters among the strains (*p* < 0.05, *F*-test).

	AFP	HFP	CFP
Viability (%)	100 ± 0 ^b^	0 ± 0 ^a^	17 ± 2 ^a^
Diameter (mm)	0.90 ± 0.10 ^a^	0.97 ± 0.21 ^a^	1.03 ± 0.25 ^a^
Wet weight (mg/fungal pellet)	0.37 ± 0.05 ^a^	0.27 ± 0.06 ^ab^	0.21 ± 0.04 ^a^
Dry mass (mg/fungal pellet)	0.04 ± 0.01 ^b^	0.03 ± 0.01 ^b^	0.01 ± 0.01 ^a^
Volume (mm^3^/fungal pellet)	0.017 ± 0.005 ^a^	0.014 ± 0.002 ^a^	0.017 ± 0.001 ^a^
Charge or zeta potential (mV)	−4.3 ± 0.6 ^a^	−2.5 ± 0.3 ^b^	−2.4 ± 0.2 ^b^
Carbon % on pellet surface	61 ± 10 ^a^	53 ± 9 ^a^	50 ± 8 ^a^
HPBI ^1^	4.7 ± 0.1 ^c^	2.1 ± 0.5 ^b^	0.7 ± 0.1 ^a^

^1^ Hydrophobicity index.

## Data Availability

Not applicable.
